# CD73-Positive Cell Spheroid Transplantation Attenuates Colonic Atrophy

**DOI:** 10.3390/pharmaceutics15030845

**Published:** 2023-03-04

**Authors:** Daisuke Hisamatsu, Natsumi Itakura, Yo Mabuchi, Rion Ozaki, Eriko Grace Suto, Yuna Naraoka, Akari Ikeda, Lisa Ito, Chihiro Akazawa

**Affiliations:** Intractable Disease Research Center, Juntendo University Graduate School of Medicine, 2-1-1 Hongo, Bunkyo-ku, Tokyo 113-8421, Japan

**Keywords:** CD73, extracellular matrix remodeling, intestinal fibroblast, mesenchymal stromal cells, three-dimensional culture, trans-anal transplantation

## Abstract

The incidence of inflammatory bowel diseases (IBD) is increasing worldwide. Mesenchymal stem/stromal cells (MSCs) have immunomodulatory functions and are a promising source for cell transplantation therapy for IBD. However, owing to their heterogeneous nature, their therapeutic efficacy in colitis is controversial and depends on the delivery route and form of transplanted cells. Cluster of differentiation (CD) 73 is widely expressed in MSCs and used to obtain a homogeneous MSC population. Herein, we determined the optimal method for MSC transplantation using CD73^+^ cells in a colitis model. mRNA sequencing analysis showed that CD73^+^ cells exhibited a downregulation of inflammatory gene expression and an upregulation of extracellular matrix-related gene expression. Furthermore, three-dimensional CD73^+^ cell spheroids showed enhanced engraftment at the injured site through the enteral route, facilitated extracellular matrix remodeling, and downregulated inflammatory gene expression in fibroblasts, leading to the attenuation of colonic atrophy. Therefore, the interaction between intestinal fibroblasts and exogenous MSCs via tissue remodeling is one mechanism that can be exploited for colitis prevention. Our results highlight that the transplantation of homogeneous cell populations with well-characterized properties is beneficial for IBD treatment.

## 1. Introduction

Inflammatory bowel diseases (IBD), including ulcerative colitis (UC) and Crohn’s disease, are intractable diseases whose incidence rate in recent years has increased up to 0.5% of the population in industrialized countries, especially Western countries [1]. Moreover, in recent years, more than 180,000 people in Asia have been reported to have UC, due to an increased Westernization of lifestyles, including food habits [2]. IBDs are caused by chronic inflammation resulting from activated immune cells; however, no fundamental therapy exists for the condition [3]. Given the immunosuppressive effects of mesenchymal stem/stromal cells (MSCs), stem cell transplantation therapy for inflammatory diseases, including IBD, has recently attracted attention [4]. Although intravenous, intraperitoneal, and anal injections are known routes for delivery of MSCs in a mouse model of dextran sulfate sodium salt (DSS)-induced colitis, their therapeutic efficacy is controversial [4,5]. MSCs form a highly heterogeneous cell population that can be isolated from either tissues or cells that attach to cultured dishes [6]. Depending on the molecules expressed on MSCs, their ability for engraftment at the transplant site and immune modulation via secreted factors may differ [7,8,9]. Moreover, recent findings have shown that spheroids derived from three-dimensional (3D) MSC cultures have enhanced the immunomodulatory function, vascularization, and multipotency via alteration of their gene expression profiles in several disease models [10,11,12]. Thus, the controversial therapeutic efficacy of the transplant route for the colitis model could be attributed to an inappropriate selection of the delivery method for the appropriate cell form.

We previously demonstrated that cluster of differentiation (CD) 73 molecules can be used to efficiently isolate cell populations with MSC characteristics from human and rodent adipose tissues [13,14]. However, the specific characteristics of CD73^+^ cells remain unclear. Moreover, the appropriate route of transplantation in the colitis model and the forms of transplanted cells, such as two-dimensional (2D) or 3D culture-derived spheroids, are unknown.

Tissue-resident fibroblasts are involved in intestinal homeostasis under both steady-state and inflammatory conditions, such as in the pathogenesis of IBD [15,16]. Under steady-state conditions, fibroblasts produce extracellular matrix (ECM)-related molecules, such as collagen I, collagen IV, fibronectin, and matrix metalloproteinases (MMPs), and maintain intestinal architecture through ECM remodeling [17]. Furthermore, fibroblasts expressing specific genes play a pivotal role in maintaining intestinal stem cells via Wnt signaling [18,19]. In contrast, single-cell analyses of patients with UC have shown that inflammation-associated fibroblasts are a heterogeneous population, divided into several subsets that can be classified according to the expression patterns of Wnt and bone morphogenetic protein (BMP) family members [20,21]. These cells act in a context-dependent manner to either promote pathogenesis or suppress inflammation. Thus, the role of fibroblasts during the development and pathogenesis of the intestinal tract remains to be clarified. However, the interactions between tissue-resident fibroblasts and exogenous MSCs during the acute or regeneration phase after MSC transplantation, as well as the functions of transplanted MSCs in intestinal homeostasis, are largely unknown.

In the present study, we hypothesized that a uniform population of CD73^+^ cells, rather than conventional heterogeneous MSCs, would have more predictive or controlled therapeutic efficacy when considering cell transplantation at the clinical stage. Our goal was to characterize CD73^+^ cells by comparing them with conventional heterogeneous MSCs, based on transcriptome analysis, and to determine the appropriate transplantation route and cell form for CD73^+^ cells in a mouse model of DSS-induced colitis. To this end, we analyzed gene and protein expressions, as well as their associations with intestinal homeostasis, in the interactions between exogenous CD73^+^ cells and fibroblasts. Our study provides insights into MSC transplantation therapy for IBD and suggests whether CD73^+^ cells are effective in this regard.

## 2. Materials and Methods

### 2.1. Experimental Animals

Male C57BL/6 NJcl (wild-type) mice (10–12 weeks old, average weight 25.16 g) were obtained from CLEA Japan, Inc. (Tokyo, Japan), and male C57BL/6-Tg (CAG-EGFP) mice (6–8 weeks old, average weight 19.78 g) were obtained from Japan SLC, Inc. (Hamamatsu, Japan). Donor cells used for transplantation were derived from CAG-EGFP mice, in order to distinguish them from cells of the wild-type recipient mice. All mice were housed in cages in a specific pathogen-free room under 12 h light/dark cycles, and received water and food ad libitum.

### 2.2. DSS-Induced Colitis Model

For the induction of colitis, 3.0% or 4.2% *w*/*v* DSS solution (MP Biomedicals, Santa Ana, CA, USA) was supplemented in drinking water and administered to wild-type mice for 5 or 7 days, with a change of water every 3 days. After DSS treatment, the mice were given regular water. Changes in body weight were measured. Mice were anesthetized with isoflurane and sacrificed for tissue sampling. In the present study, two different DSS concentrations (3.0% and 4.2%) were used to account for variations. The concentration at which weight loss was observed in more than 90% of the individuals in pilot experiments was adopted for each lot of DSS.

### 2.3. Isolation and Intravenous Administration of CD73-Positive Cells

CD73^+^ cells were isolated from the subcutaneous fat tissue of mice, as previously described [13]. The cells were washed with Hank’s balanced salt solution (HBSS, Nacalai Tesque, Kyoto, Japan) and 4% fetal bovine serum (FBS, Thermo Fisher Scientific, Waltham, MA, USA) before staining with a fluorescence-conjugated antibody cocktail for 30 min on ice. The following antibodies were used: Ter119 (TER119), CD45 (30-F11), CD31 (390), and CD73 (TY11.8) (BD Biosciences, Franklin Lakes, NJ, USA). CD73^+^ cells were defined as Ter119-CD45-CD31-CD73^+^ live cells, and were sorted on the FACS AriaII (BD Biosciences). All experiments were analyzed using the FlowJo software, version 10.6.1 (TreeStar, Ashland, OR, USA). CD73^+^ cells were cultured in an MSC medium, as previously described, and were never passaged more than five times [13]. After expanding the culture, CD73^+^ cells (3.0 × 10^5^ cells in 300 µL phosphate-buffered saline; PBS) were administered to the mice by intravenous injection 1 and 5 days after DSS treatment.

### 2.4. Spheroid Formation and Trans-anal Transplantation

Exactly 14 days after sorting, CD73^+^ cells were cultured for 2 days in EZSPHERE^®^ (AGC Techno Glass, Shizuoka, Japan), in order to generate spheroids. Spheroids were collected and suspended in PBS. Cells (1.0 × 10^6^ cells in 200 μL of PBS) were transplanted via enteral administration 0 and 5 days after DSS treatment. Trans-anal transplantation was performed as previously described, with slight modifications [22]. The cell suspension was infused into the murine colonic lumen by using a thin, flexible catheter (diameter: 2.1 mm) or a stainless steel needle (diameter: 1.9 mm). After transplantation, the mice were housed as usual, and colon tissues were collected and analyzed by flow cytometry. Control mice received an equal volume of PBS, or a solution of 0.01 mg/mL 5-aminosalicylic acid (5-ASA; Zeria Pharmaceutical Co., Ltd., Tokyo, Japan). The concentration of 5-ASA (100 mg/kg) was based on a previous paper, and 5-ASA was administered enterally in the same manner as the spheroids [23].

### 2.5. Flow Cytometry

For analysis of colonic tissue, after measuring the colon length, the distal colon, 2 cm above the rectum, was cut longitudinally and washed with PBS. The tissue was minced and digested with 2 mg/mL collagenase (FUJI-FILM Wako Pure Chemical, Osaka, Japan) solution containing 10 μM DNase I (Sigma-Aldrich, St. Louis, MO, USA) in Dulbecco’s modified Eagle medium (DMEM)–GlutaMAX (Thermo Fisher Scientific), with shaking for 1 h at 37 °C. The digested tissue was filtered through a 70 μm cell strainer (CORNING, Corning, NY, USA), pelleted by centrifugation at 1800× *g* for 5 min at 4 °C, and resuspended in HBSS containing 2% FBS, 10 nM HEPES, and 1% penicillin/streptomycin (Thermo Fisher Scientific). The cell suspensions were stained with an antibody cocktail for 30 min on ice. The following antibodies were used: CD45 (30-F11), from BD Biosciences, and CD11b (M1/70) and F4/80 (BM8), from BioLegend (San Diego, CA, USA). The following antibodies were used for the analysis of MSC markers: CD73 (TY11.8), CD44 (1M7), Sca-1 (E13-161.7), and CD140a (APA5), from BD Biosciences, and CD90.2 (30-H12), from BioLegend. Data acquisition was performed using FACS AriaII (BD Biosciences), and all analyses were carried out using FlowJo software, version 10.6.1 (TreeStar).

### 2.6. Collection of Conditioned Media (CM)

CD73^+^ 2D or 3D cultures were initiated with the same number (1.0 × 10^7^) of cells and grown in the same MSC medium for 2 days. The medium was replaced with fresh MSC medium, without FBS, before sampling, and was collected 24 h later.

### 2.7. Cytometric Bead Array (CBA)

The level of cytokines in the CM was determined using CBA (BD Biosciences), according to the manufacturer’s instructions. All samples were diluted 1:2 and analyzed in duplicates. All data were recorded on a BD FACS Verse Flow Cytometer (BD Biosciences) and analyzed using FCAP Array Software v3.0 (BD Biosciences).

### 2.8. Cell Proliferation and Contraction Assays

For growth curves, murine embryonic fibroblasts (1.0 × 10^3^ cells) were seeded in 48-well plates and cultured in DMEM medium (Nacalai Tesque) with 10% FBS, 1% penicillin/streptomycin, and 2 mM L-glutamine (Nacalai Tesque), supplemented with 50% CM after 1 day for up to 2 weeks, and the numbers of live and dead cells were then determined at the indicated times. A collagen-based cell contraction assay of murine embryonic fibroblasts (1.0 × 10^5^ cells) was performed in 48-well plates, according to the manufacturer’s instructions (Cell Biolabs, Inc., San Diego, CA, USA). Before releasing the stressed matrix, the culture medium was replaced with fresh medium, containing 50% CM. The collagen gel size was evaluated using the BZ-H4M software, version 1.4.1.1 of the BZ-X710 microscope (Keyence, Osaka, Japan).

### 2.9. Quantitative Real-Time Polymerase Chain Reaction (qPCR)

Total RNA extraction and qPCR were performed as described previously [13]. Gene expression was normalized to *Gapdh* expression. Primer sequences used for qPCR are listed in Table S1.

### 2.10. Acquisition of Clinical Samples

Human adipose tissue samples were provided from surplus tissues derived from breast reconstructive and liposuction surgeries, from patients without underlying diseases such as diabetes (three individuals). Human adipose-derived CD73^+^ cells and conventional heterogeneous MSCs (cMSCs) were established from the same samples.

### 2.11. mRNA Sequencing (mRNA-Seq)

CD73^+^ cells and cMSCs were isolated from human adipose tissue and cultured as previously described [14]. Cell suspensions were incubated with APC-conjugated anti-CD73 (BioLegend) for single-color staining. To distinguish live cells from dead cells, the cells were suspended in a propidium iodide solution and sorted using FACS Aria II (BD Biosciences). All experiments were analyzed using the FlowJo software, version 10.6.1 (TreeStar). Total RNA was extracted from these cells using the RNeasy Mini kit (Qiagen, Hilden, Germany), according to the manufacturer’s instructions. Extracted RNA samples were then sent to Macrogen (Tokyo, Japan). The sequencing library was prepared using the SMART-Seq v4 Ultra-Low Input RNA kit (Takara, Kusatsu, Japan), and mRNA-seq, on the Illumina NovaSeq 6000 (paired-end, 150 bp), was performed by Macrogen.

To generate a sequence alignment, quality control and preprocessing of FASTQ data were performed using fastp v.0.20.1 (https://github.com/OpenGene/fastp (accessed on 19 April 2021)) [24], and the transcript was quantified using Salmon (https://combine-lab.github.io/salmon/ (accessed on 19 April 2021)) [25]. Data analyses and visualizations were conducted in the RStudio environment. The gene ontology (GO) annotation was determined using the DAVID online software, version 2021 (http://david.abcc.ncifcrf.gov (accessed on 11 December 2021)). To compare the enriched genes between cMSCs and CD73^+^ cells, we used the GSEA software, version 4.2.3 (Broad Institute, Cambridge, MA, USA).

### 2.12. Histological Analyses

Colonic tissues were collected from mice as previously described [22]. The histological score after DSS treatment was defined using the following criteria [26,27]: mucosal architecture (0–3), immune cell infiltration (0–3), muscle layer thickness (0–3), depletion of goblet cells (0–1), and crypt abscess (0–1). Hematoxylin and eosin (H&E) and immunofluorescence staining was performed as previously described [14]. The colon length was measured using the measuring tool in Adobe Illustrator, and was then converted to centimeters.

### 2.13. Statistical Analysis

A minimum number of three independent experiments was included in each statistical analysis. Statistical significance was determined by using the Tukey’s test and Welch’s *t*-test, and was set at *p* < 0.05. All data are shown as mean ± standard error of mean (SEM). Analyses were performed using the statistical programming language R, version 4.0.3 (2020-10-10).

## 3. Results

### 3.1. Intravenous Injection of CD73^+^ Cells Attenuates Tissue Destruction in DSS-Induced Colitis

**Figure 1:**
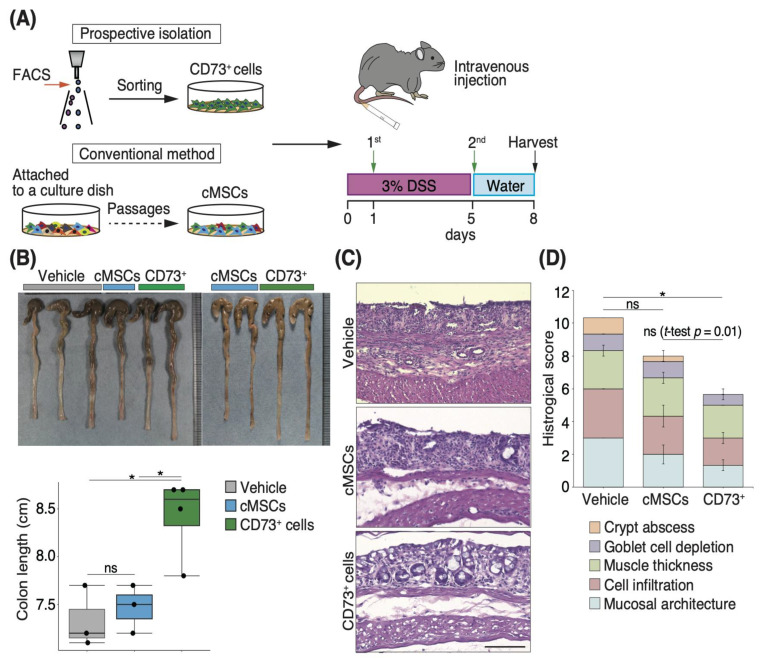
Intravenous injection of CD73^+^ cells attenuates the destruction of intestinal structure in dextran sulfate sodium salt (DSS)-induced colitis. (**A**) Schematic diagram showing the isolation method and transplantation schedule of CD73^+^ cells and conventional heterogeneous mesenchymal stem cells (cMSCs). (**B**) Representative images of the colon after DSS-induced colitis. Lower graph shows the colon length 8 days after DSS administration. Vehicle, phosphate-buffered saline (PBS)-treated group; cMSCs, cMSC-transplanted group; CD73^+^, CD73^+^ cell-transplanted group. *n* = 10 mice from 2 independent experiments. Two mice in the PBS-treated group and one mouse in the cMSC-transplanted group died before analysis. (**C**) Representative images of hematoxylin and eosin (H&E) staining of the injured colonic tissue. Scale bar: 100 μm. (**D**) Graph showing histological score based on H&E staining of the colonic tissue. The colors represent the following criteria: mucosal architecture (0–3), immune cell infiltration (0–3), muscle layer thickness (0–3), depletion of goblet cells (0–1), and crypt abscess (0–1). Statistical significance was determined using the Tukey’s test and Welch’s *t*-test. Data are shown as mean ± standard error of mean (SEM). * *p* < 0.05; ns, not significant.

To investigate the efficacy of CD73^+^ cell transplantation in a mouse model of DSS-induced colitis, we initially isolated CD45-CD31-Ter119-CD73^+^ (CD73^+^) cells from the subcutaneous white adipose tissue of mice, using fluorescence-activated cell sorting (FACS), and compared them with cMSCs attached to the culture dish (Figure S1 and Figure 1A). In the present study, given the relevance of therapeutic strategies at an early stage, before the colitis worsens, the first intravenous injection was administered a day after the administration of DSS in drinking water, and the second dose was administered on day 5 (Figure 1A). After 3 days of recovery with regular water following DSS exposure, the colon length was measured. Although there was no significant difference between the cMSC-transplanted and PBS-treated groups, the CD73^+^ cell-transplanted group had a significantly longer colon length than the PBS-treated and cMSC-transplanted groups (*p* = 0.01 and *p* = 0.02, respectively; Figure 1B). Based on the colitis-associated histological score [26,27], we evaluated the colon mucosa after H&E staining, using the following criteria: mucosal architecture (0–3), immune cell infiltration (0–3), muscle layer thickness (0–3), depletion of goblet cells (0–1), and crypt abscess (0–1). The overall score of the CD73^+^ cell-transplanted group was lower than those of the cMSC-transplanted and PBS-treated groups (mean scores: CD73^+^ 5.6, cMSC 8.0, and PBS 10.3; Figure 1C,D). Specifically, the scores for the mucosal architecture and crypt abscess were significantly lower in the CD73^+^ cell-transplanted group than in the PBS-treated group (*p* = 0.05 and *p* = 0.02, respectively). Altogether, these results suggest that an intravenous injection of CD73^+^ cells has more regenerative potency than an intravenous injection of cMSCs in DSS-induced colitis.

### 3.2. ECM-Related Genes Are Enriched in the CD73^+^ Cell Population

**Figure 2:**
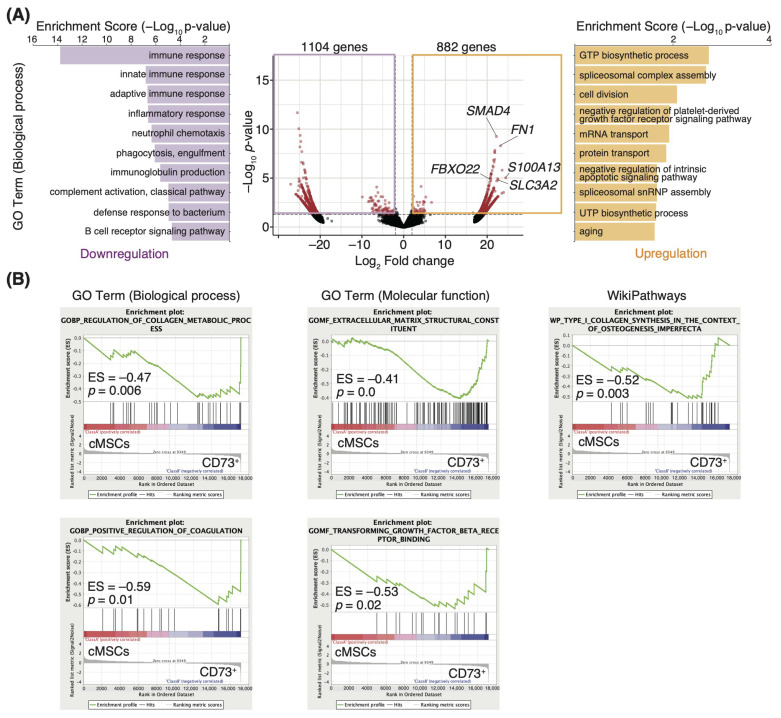
CD73^+^ cells exhibit upregulated expression of genes, related to extracellular matrix (ECM) remodeling and the downregulated expression of genes related to the immune response compared with cMSCs. (**A**) mRNA-seq-based measurements of gene expression changes in CD73^+^ cells compared with cMSCs, isolated from human adipose tissues. *n* = 3 specimens. Volcano plots showing differentially expressed genes (log2 fold-change > 1 or <–1 (vertical dashed lines), and *p* < 0.05 (horizontal dashed line)). Graphs showing the top 10 enriched gene ontology (GO) biological process terms. The left panel shows the downregulated genes, and the right panel shows the upregulated genes in CD73^+^ cells. (**B**) Gene set enrichment analysis of CD73^+^ cells and cMSCs. ES, enrichment score.

Since previous studies showed that the CD73^+^ cell population has similar phenotypes in mouse and human MSCs [13,28,29], we performed a comprehensive transcriptome analysis using human cells, considering the clinical application of CD73^+^ cells. To characterize the high regenerative efficacy of CD73^+^ cells, we compared gene expression profiles between cMSCs and CD73^+^ cells isolated from human adipose tissues (Figure S2), and identified the significantly differentially expressed genes; a total of 882 upregulated and 1104 downregulated genes (log_2_ fold change >1 or <−1, *p* < 0.05; Figure 2A) were identified in the CD73^+^ cells. The top 10 GO biological process terms for the upregulated genes in CD73^+^ cells, as determined by the GO enrichment analysis, included “GTP biosynthetic process”, “spliceosomal complex assembly”, “cell division”, “negative regulation of platelet-derived growth factor receptor signaling pathway”, and “mRNA transport.” In contrast, the terms for the downregulated genes were “immune response”, “innate immune response”, “adaptive immune response”, “inflammatory response”, and “neutrophil chemotaxis” (Figure 2A). We also found that *SMAD4*, fibronectin 1 *(FN1)*, *S100A13*, *FBXO22*, and *SLC3A2* were considerably upregulated in CD73^+^ cells (adjusted *p*-values < 0.05; Figure 2A). These genes are involved in integrin signaling and angiogenesis [30,31,32,33,34]. These results are consistent with the results of the gene set enrichment analysis (GSEA), in which the genes enriched in CD73^+^ cells were related to the “regulation of collagen metabolic process”, “positive regulation of coagulation”, “extracellular matrix structural constituent”, and “transforming growth factor beta receptor binding” (Figure 2B). Furthermore, intravenously injected cells rarely engrafted into the colon (Figure S3), consistent with the results of a previous study, in which transplanted cells engrafted into the lungs [35]. These results suggest that the regenerative efficacy of transplantation is due to the paracrine effect of secretory factors from engrafted cells at the transplant site. Therefore, we focused on the terms associated with paracrine signaling in our GO analysis results, and “Wnt signaling pathway (GO:0016055)” was identified as being overrepresented in the genes upregulated in CD73^+^ cells (enrichment score: 1.22, *p* = 0.06).

### 3.3. Three-Dimensional Spheroids of CD73^+^ Cells Have More Engraftment Potential than Two-Dimensional Cells in Trans-Anal Transplantation

Considering that the genes related to cell adhesion, such as those involved in integrin signaling, were upregulated in CD73^+^ cells (Figure 2B), we hypothesized that CD73^+^ cells are suitable for engraftment to injured sites and attempted trans-anal transplantation, which provides more access to the injured site in the distal colon than intravenous injection. Moreover, 3D culture-derived spheroid transplantation is associated with enhanced engraftment when compared with 2D cell transplantation [36]. In particular, 3D culture-derived intestinal organoids engraft in the injured colonic lumen after trans-anal administration [22]. We first optimized the delivery method and compared a thin, flexible catheter with a 2.1 mm diameter to a stainless steel needle with a 1.9 mm diameter, and found that using the needle obtained a higher survival rate than the catheter (Figure S4A,B). We further investigated the differences in the engraftment rate of transplanted cells via the enteral route using a stainless steel needle, depending on the type of transplanted cells. On day 2 after green fluorescent protein (GFP)-positive cells were transplanted, the positivity rate was evaluated by FACS analysis of the distal colon tissue. We observed a significantly higher engraftment rate after spheroid transplantation than after 2D cell transplantation (*p* = 0.01; Figure S5A,B).

### 3.4. Transplantation of CD73^+^ Cell Spheroids onto the Colonic Lumen Prevents Mucosal Atrophy

**Figure 3:**
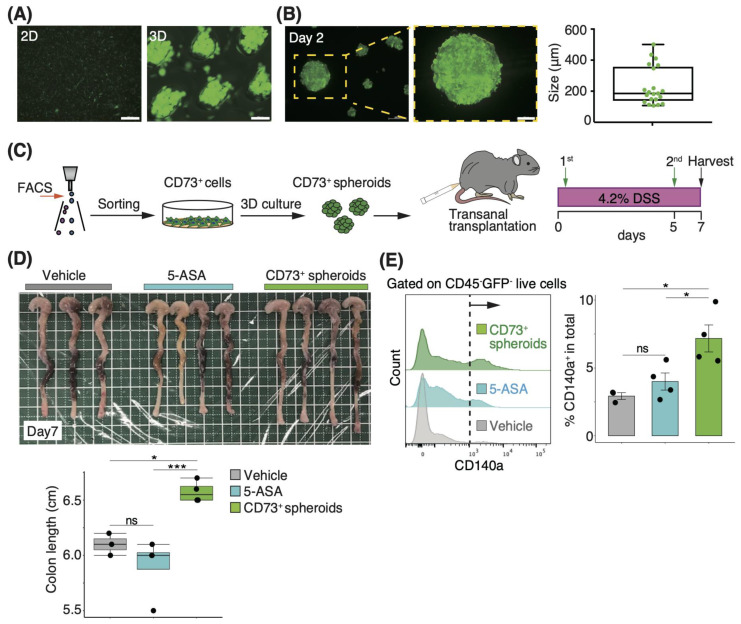
Transplantation of CD73^+^ cell spheroids onto the colonic lumen prevents mucosal atrophy. (**A**) Representative images of CD73^+^ cells and 3D culture-derived spheroids. Scale bars: 2D, 500 μm, 3D, 200 μm. (**B**) Representative image of a single spheroid. Graph showing the spheroid size after 2 days of culture. Scale bar: 100 μm. *n* = 21 spheroids from 2 independent experiments. (**C**) Schematic representation of the spheroid transplantation strategy via trans-anal transplantation. (**D**) Representative images of the colon on day 7 after DSS treatment. Graphs showing the colon length. Vehicle, PBS-treated group; 5-aminosalicylic acid (5-ASA), 5-ASA-treated group; CD73^+^ spheroids, CD73^+^ cell spheroid-transplanted group. *n* = 3 (PBS), *n* = 4 (5-ASA), *n* = 4 (CD73^+^ spheroids) mice. (**E**) Histogram showing GFP-CD45-CD140a^+^ single live cells in each group, as indicated in the panel by FACS analysis. The dashed line denotes the threshold of the positive rate, and the graphs on the right show the quantitative data on day 7. Statistical significance was determined using the Tukey’s test. Data are shown as mean ± SEM. * *p* < 0.05; *** *p* < 0.005; ns, not significant.

We found the trans-anal transplantation of spheroids to be an effective delivery method for CD73^+^ cells, as indicated by the engraftment rate, but the therapeutic efficacy of this method on DSS-induced colitis is unknown. We prepared CD73^+^ cell-derived spheroids of 185.1 μm diameter (median) through 2 days of 3D culture (Figure 3A,B). To examine the effect of transplantation in the acute phase, we transferred these spheroids via the trans-anal route on days 0 and 5 of DSS administration, and then measured the colon length on day 7 after DSS administration (Figure 3C). The colon length in the CD73^+^ spheroid-transplanted group was significantly higher than that in the PBS- and 5-ASA-treated groups (5-ASA is used clinically for treating UC [37]) on day 7 after DSS administration (*p* = 0.02 and *p* < 0.005, respectively; Figure 3D). Moreover, the proportion of endogenous GFP-CD45-CD140a^+^ fibroblasts in colonic tissue was significantly higher in the CD73^+^ spheroid-transplanted group than in the PBS- and 5-ASA-treated groups on day 7 after DSS administration (*p* = 0.01 and *p* = 0.04, respectively; Figure 3E). These results suggest that CD73^+^ cell spheroid transplantation via the trans-anal route prevents the development of colitis atrophy during the acute phase.

### 3.5. Three-Dimensional Spheroids Enhance the Properties of CD73^+^ Cells

**Figure 4:**
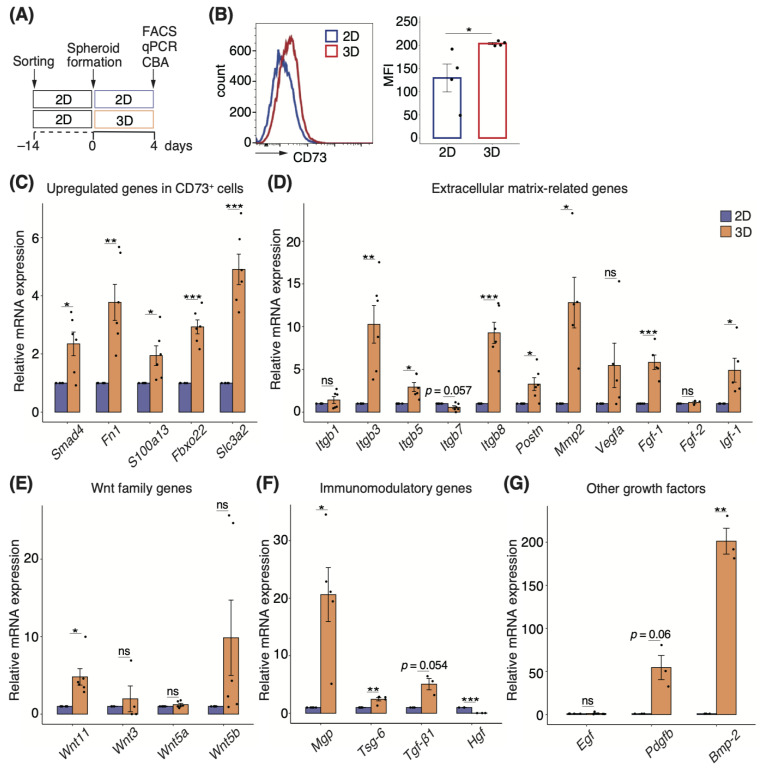
Three-dimensional spheroids enhance the properties of CD73^+^ cells. (**A**) Schematic representation of the comparative analysis indicated in the panel, for two-dimensional (2D) and three-dimensional (3D) cultures of CD73^+^ cells. (**B**) Histogram showing the frequency of CD73^+^ cells after 4 days of culture, as determined using FACS analysis. The graph shows the quantification of mean fluorescence intensity (MFI). *n* = 4. Relative mRNA expression of the upregulated genes identified by transcriptome analysis in CD73^+^ cells (**C**), including extracellular matrix-related genes (**D**), Wnt family genes (**E**), immunomodulatory genes (**F**), and growth factor genes of interest (**G**). *n* = 4–6. Statistical significance was determined using the Welch’s *t*-test. Data are shown as mean ± SEM. * *p* < 0.05; ** *p* < 0.01; *** *p* < 0.005; ns, not significant.

To determine the mechanism underlying the reduction of colitis in the CD73^+^ cell spheroid-transplanted group, we characterized different cell forms in terms of gene and protein expression (Figure 4A). We first examined protein expression using FACS analysis and found that CD73 expression was significantly higher in 3D cultures (*p* = 0.049; Figure 4B). Further analysis of other MSC markers revealed no difference in the expression of CD44 and CD90, but the expression of CD140a and Sca-1 was significantly lower in 3D cultures than in 2D cultures (*p* = 0.02 and *p* = 0.01, respectively; Figure S6A,B).

Next, we analyzed the significantly upregulated genes in CD73^+^ cell populations compared with cMSCs (Figure 2A). The relative expression of *Smad4*, *Fn1*, *S100a13*, *Fbxo22*, and *Slc3a1* was significantly higher in CD73^+^ cell spheroids than in 2D cells (Figure 4C). Moreover, because gene sets related to ECM, such as integrin signaling and angiogenesis, were enriched in CD73^+^ cells (Figure 2B), we investigated the expression of genes involved in the same, and found that *Itgb3*, *Itgb5*, *Itgb8*, periostin (*Postn*), *Mmp2*, *Fgf-1*, and *Igf-1* were significantly upregulated in 3D cultures (Figure 4D). Although the Wnt family genes were slightly enriched in CD73^+^ cells, *Wnt11* expression was significantly upregulated in spheroids (Figure 4E). We investigated gene expression by focusing on the immunomodulatory mechanisms of MSCs. mRNA expression levels of matrix Gla protein (*MGP*) and tumor necrosis factor-α-stimulated gene 6 (*TSG-6*) were significantly upregulated, whereas hepatocyte growth factor (*HGF*) expression was significantly downregulated in 3D cultures (Figure 4F). The expression of another growth factor, *Bmp-2*, which promotes osteogenic differentiation, was significantly upregulated in 3D cultures (Figure 4G).

**Figure 5:**
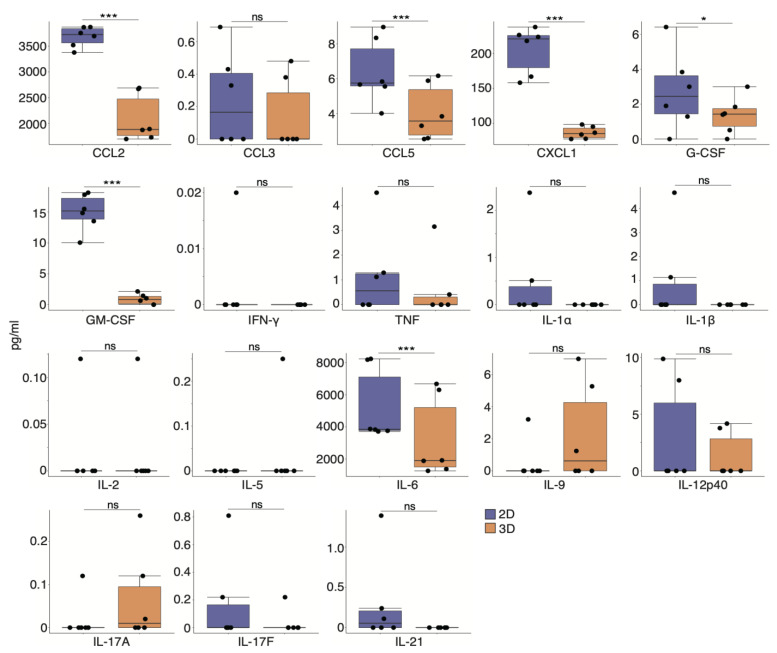
Inflammatory cytokine levels are downregulated by secretory factors of CD73^+^ cell spheroids. Cytokines in the culture supernatants of 2D and 3D cells were quantified using a cytometric bead array. *n* = 6. Statistical significance was determined using the Welch’s *t*-test. Data are shown as mean ± SEM. * *p* < 0.05; *** *p* < 0.005; ns, not significant.

To further characterize the secretory profile of CD73^+^ cell spheroids, we carried out a CBA of various cytokines related to inflammatory signatures that were downregulated in CD73^+^ cells compared with cMSCs (Figure 2A). A comprehensive analysis of culture supernatants revealed that the levels of CCL2, CCL5, CXCL1, G-CSF, GM-CSF, and interleukin (IL)-6 were significantly lower in 3D cultures than in 2D cultures (Figure 5).

### 3.6. Secretory Factors of CD73^+^ Cell Spheroids Induce Alterations of ECM Remodeling in Fibroblasts

**Figure 6:**
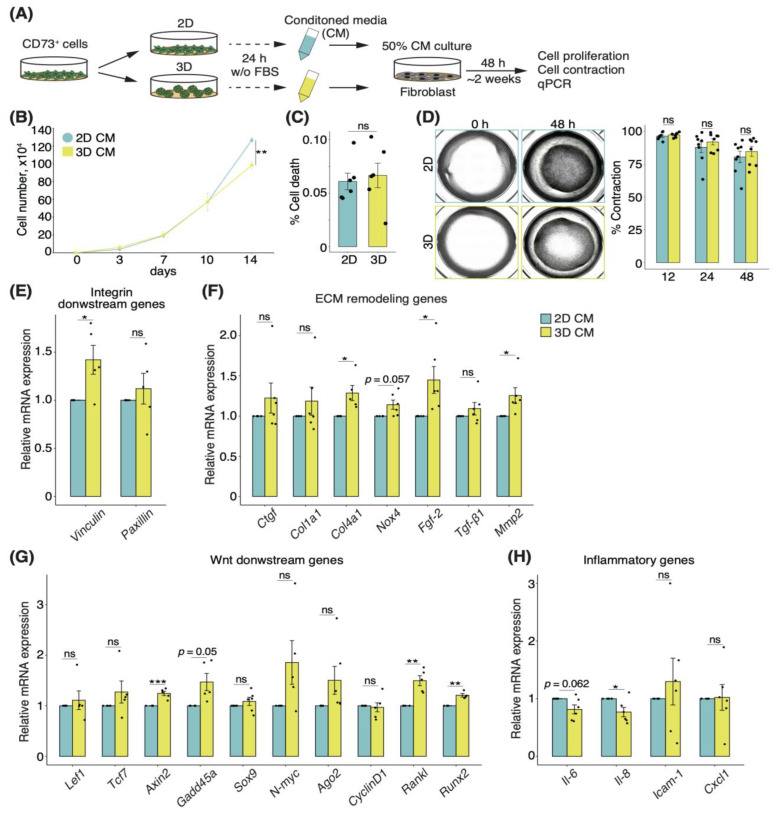
Secretory factors of CD73^+^ cell spheroids induce an alteration of ECM remodeling in fibroblasts. (**A**) Schematic representation of how the conditioned media (CM) were obtained and used to treat the fibroblasts. (**B**) Graph showing the number of cells at each time point. *n* = 9 from 3 independent experiments. (**C**) Percentage of cell death at 14 days of culture. *n* = 9 from 3 independent experiments. (**D**) Representative images of cell contraction assay after 48 h. The graph shows the contraction rate at each time point. *n* = 3 per 3 supernatants. Relative mRNA expression of integrin-downstream genes (**E**), ECM remodeling genes (**F**), Wnt-downstream genes (**G**), and inflammatory genes (**H**) in fibroblasts after 48 h of culture, in each CM. *n* = 6 from 2 independent experiments. Statistical significance was determined using the Welch’s *t*-test. Data are shown as mean ± SEM. * *p* < 0.05; ** *p* < 0.01; *** *p* < 0.005; ns, not significant.

Although we showed that trans-anal transplantation of CD73^+^ spheroids attenuated DSS-induced colonic atrophy and increased the proportion of tissue-resident fibroblasts (Figure 3D,E), the molecular mechanism remains unclear. Motivated by the fact that stromal cells, including fibroblasts with myofiber features, are associated with UC [16], we investigated the effects of alterations in fibroblasts by secretory factors of CD73^+^ cell spheroids. To this end, we prepared the CM of CD73^+^ 2D cells and spheroids without FBS, cultured fibroblasts in regular medium supplemented with 50% CM, and analyzed cell proliferation, contraction, and gene expression at the indicated time points (Figure 6A). On day 14, the growth assay showed a significantly lower proliferation of fibroblasts cultured in the CM of 3D cultures when compared to those cultured in the CM of 2D cultures (fold-change 0.78, *p* < 0.01; Figure 6B). Furthermore, the rate of cell death remained unchanged (Figure 6C), suggesting that the secretory factors of the culture supernatant affected the proliferative capacity. In contrast, the cell contraction assay showed no difference between the CM of 2D and 3D cultures (Figure 6D).

Finally, we investigated the effect of both 3D and 2D cultures, in the CM, on fibroblasts. The expression levels of *VCL,* which encodes vinculin and is an integrin-downstream gene, as well as the ECM remodeling-related genes *Col4a1*, *Mmp2*, and *Fgf-2,* were upregulated in the fibroblasts cultured in the CM of CD73^+^ cell spheroids (Figure 6E,F). Moreover, the expression levels of Wnt-downstream genes, such as *Axin2*, *Gadd45a*, *Rankl*, and *Runx2*, were upregulated in the fibroblasts cultured in the CM of 3D cultures (Figure 6G). In contrast, *Il-6* and *Il-8* expression levels were downregulated in the fibroblasts cultured in the CM of 3D cultures (Figure 6H). Altogether, these data suggest that the secretory factors of CD73^+^ cell spheroids change the gene expression profile of fibroblasts and specifically induce ECM remodeling and the suppression of inflammation.

## 4. Discussion

**Figure 7:**
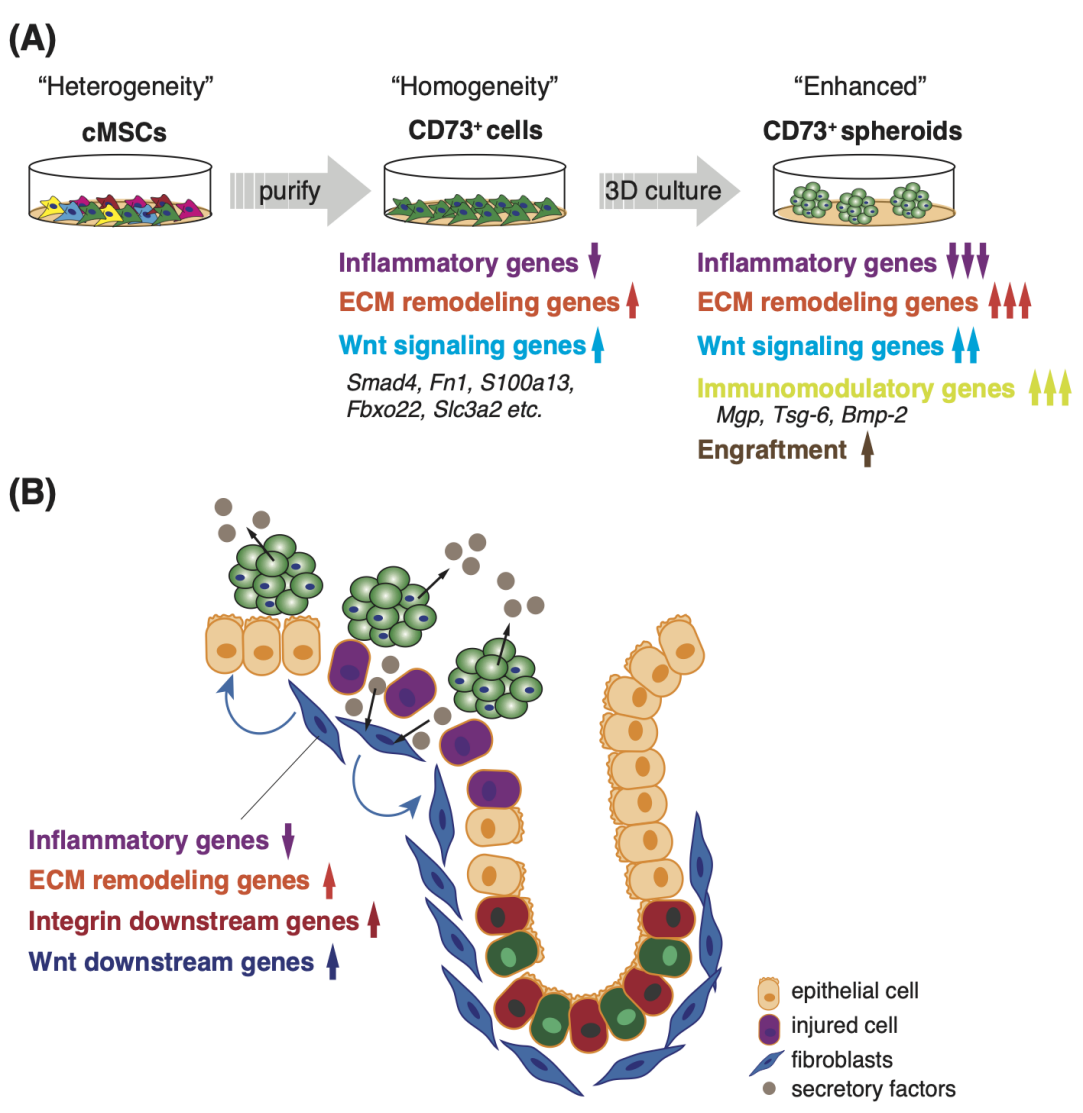
Proposed model for interaction between intestinal fibroblasts and transplanted CD73^+^ cell spheroids in DSS-induced colitis. (**A**) Compared with conventional heterogeneous MSCs, the homogeneous CD73^+^ cell population, purified by prospective isolation, exhibits characteristic gene expression, including the downregulation of inflammatory factors and the upregulation of ECM remodeling- and Wnt-signaling-related genes. Moreover, 3D culture-derived spheroids of CD73^+^ cells enhance these characteristics, and induce the upregulation of immunomodulatory factor expression, suggesting enhanced engraftment into colon tissues after DSS treatment. (**B**) Transplanted CD73^+^ cell spheroids attenuate colitis by modifying the phenotype of the tissue-resident fibroblasts, including downregulating the expression of inflammatory factors, and upregulating the expression of ECM remodeling- and Wnt signaling-related genes.

In this study, we demonstrated that CD73^+^ cells, prospectively isolated from adipose tissue, exhibited a reduced inflammatory signature expression and elevated ECM remodeling-related gene expression, including those involved in the Wnt signaling pathway, compared with conventionally heterogeneous MSCs. Furthermore, 3D culture-derived spheroids of CD73^+^ cells showed enhanced cellular characteristics and an upregulated expression of immunomodulatory genes, such as *Mgp*, *Tsg-6*, and *Bmp-2*, compared with 2D cells (Figure 7A). In addition, the levels of CCL2 and CCL5, which are involved in fibroblast proliferation, were reduced in the culture supernatant after 3D culture [38]. The trans-anal transplantation of CD73^+^ cell spheroids showed an enhanced engraftment onto the colonic lumen compared with the 2D cells. Moreover, the proportion of tissue-resident CD140a^+^ fibroblasts increased in the CD73^+^ cell spheroid transplantation group. These data imply that the interaction between the transplanted cells and fibroblasts attenuated colonic atrophy in the acute phase. Indeed, we found that the culture supernatant of CD73^+^ cell spheroids altered the expression of ECM remodeling-related genes and inflammatory cytokines in fibroblasts. Collectively, trans-anal transplantation of spheroids is appropriate for the clinical application of homogenous CD73^+^ cells in treating IBD. One mechanism underlying the mitigation of colonic atrophy might be the enhancement of remodeling in tissue-resident fibroblasts, by secreted factors derived from the engrafted CD73^+^ cell spheroids (Figure 7B).

Fibronectin, whose expression was upregulated in CD73^+^ cells and its spheroids (Figure 2A and Figure 4C), is a key regulator of ECM remodeling through its involvement in cell proliferation and adhesion signaling [17]. Fibronectin is abundant in the colonic epithelium under steady-state conditions, but its expression is elevated in the colonic tissues of patients with IBD, as well as DSS-induced colitis mice [39]. Epithelium-derived fibronectin enhances cell adhesion and wound healing [40]. SLC3A2, also known as a heavy chain of CD98 (CD98hc), regulates integrin signaling, which is pivotal for ECM remodeling [34,41]. This factor contributes to integrin-dependent cell migration and protection from cell death in fibroblasts, and modulates intestinal homeostasis, especially through its expression in epithelial cells under inflammatory conditions [42]. Altogether, these previous studies and our findings suggest that the effectiveness of CD73^+^ cell spheroids in attenuating colonic atrophy is attributable to the interaction or synergistic effect among exogenously transplanted cells, fibroblasts, and epithelial cells.

S100A13 and FBXO22 are involved in angiogenesis [33,43], which is also a crucial process in wound healing that regulates ECM remodeling [44]. In particular, S100A13, a small Ca^2+^-binding protein, is thought to regulate the co-secretion of FGF-1, a potent angiogenic factor, or they are thought to enhance each other’s expression [32,45]. *S100a13* and *Fgf-1* expression was upregulated in CD73^+^ cell spheroids (Figure 4C,D), suggesting that S100A13 and FGF-1 may work in tandem to promote angiogenesis at injured sites.

Given the paracrine effects of engrafted CD73^+^ cell spheroids, we focused on secretory factors, including Wnt-signaling molecules. Previous studies have shown that fibroblasts that release Wnt and POSTN are essential for supporting intestinal stem cell renewal [19]. However, upon inflammation, such as in colitis, activated fibroblasts that exhibit inflammatory signatures impair epithelial regeneration, and further exacerbate pathogenesis [20,21]. We demonstrated that the transplantation of CD73^+^ cell spheroids increased the proportion of CD140a^+^ fibroblasts present in the colonic tissue (Figure 3E), and secretory factors released by CD73^+^ cell spheroids partially led to the activation of the Wnt signaling pathway and the downregulation of inflammatory factors in fibroblasts (Figure 6G,H). Wnt signaling drives fibrosis via molecular cross-talk with TGF-β signaling [46]. Fibrosis impacts tissue repair and pathogenesis both positively and negatively [47]. Fibroblasts cultured in the supernatant of CD73^+^ cell spheroids showed an increased expression of *Col4a1* and a lowered proliferative ability when compared to those cultured in the 2D supernatant (Figure 6B,G). Collectively, these data suggest the activation of Wnt signaling and ECM remodeling in the fibroblasts. We speculate that CD73^+^ cell spheroids, with an elevated expression of Wnt signaling pathway-related genes and *POSTN,* replace fibroblasts that maintain intestinal homeostasis at the inflamed site, suppress inflammation, and promote early repair or protect the niche, including fibroblasts present in the steady state. However, the effect of CD73^+^ cell spheroids on inflammation-associated fibroblasts and the role of activated CD140a^+^ fibroblasts remain unclear. Recent findings indicate that IL-11^+^ fibroblasts promote colorectal cancer and acute colitis [48]. Using an *Il11* reporter mouse to perform CD73^+^ cell transplantation experiments will clarify its effects on inflammatory fibroblasts.

In the present study, we compared the therapeutic efficacy of spheroid transplantation with that of 5-ASA, which is prescribed to more than 80% of patients with UC as a first-line treatment [37]. Although the benefits of 5-ASA are mainly due to its anti-inflammatory effects [49], the trans-anal administration of 5-ASA did not appear to improve colon length or affect fibroblasts’ properties during the acute phase of DSS-induced colitis (Figure 3D,E). Previous studies showed that even when 5-ASA was orally administered once daily, from day 1 to day 10, insufficient therapeutic effects were observed in the acute phase of the DSS-induced colitis model on days 5 to 7 [50,51]. During the wound-healing process, an inflammatory state has both negative and positive impacts [52]. MSCs are known to be a source of immunomodulatory factors, which are pivotal for tissue repair in clinical applications [53,54]. TSG-6, an immunomodulatory factor, improves myocardial infarction through its anti-inflammatory effects [55]. Furthermore, MSC aggregation augments TSG-6 expression [10], which is consistent with our results (Figure 4F). In addition, MGP, whose expression was upregulated in CD73^+^ cell spheroids (Figure 4F), reportedly ameliorates pathogenesis in a mouse model of Crohn’s disease through its MSC-derived immunomodulatory function that suppresses T cell proliferation and cytokine production [56]. Overall, we suggest that CD73^+^ cell spheroids, which express high levels of immunomodulatory factors that regulate the balance of the immune system, may be more protective than 5-ASA, which has a strong anti-inflammatory effect, in the acute phase of tissue repair.

The present study has some limitations. First, it is unknown as to whether CD73^+^ cell spheroid transplantation has potential side effects. Inhibition of CD73 activity or gene silencing in tumor cells is reported to attenuate tumorigenesis [57,58]. Additionally, CD73 is highly expressed in solid tumors and is reported to facilitate colitis-associated tumorigenesis [59,60]. These findings suggest that transplantation of CD73^+^ cells could lead to tumor formation. However, the promotion of colitis-associated tumorigenesis by CD73, reported by Liu et al., was observed in a chronic inflammatory model generated following three cycles of treatment with azoxymethane, a genotoxic colonic carcinogen, as well as DSS administration for 3 weeks [60], which is different from the acute colitis model used in the present study. Moreover, transplanted MSCs disappear after temporary engraftment [61], but we will further investigate whether CD73^+^ cell spheroids remain at the injured site for a long time using longitudinal analysis, considering the potential clinical application of MSC transplantation. Second, the function of CD73 remains unclear. CD73 is a glycosyl phosphatidylinositol-anchored membrane protein and one of the ectonucleotidases that metabolize extracellular adenosine triphosphate (ATP) [62]. CD73 converts 5′-adenosine monophosphate (AMP) to adenosine, which contributes to immunosurveillance in various tumor microenvironments [62]. Our data also showed that the expression of gene sets related to metabolism, such as the “GTP biosynthetic process” and “UTP biosynthetic process”, was enriched in CD73^+^ cells (Figure 2A), suggesting that the transplanted CD73^+^ cell spheroids have a distinct adenosine-mediated role, independent of the ECM remodeling pathway reported in the present study. Adenosine in colitis regulates inflammatory responses, such as epithelial hyperpermeability, but it elicits different phenotypes depending on the receptor subset [63]. In addition, CD73 and adenosine receptors are widely expressed on tumor cells and immune cells, including regulatory T cells, which play a role in suppressing inflammation, and play context-dependent roles in various cell types [59]. Specifically, primary colorectal cancers associated with IBD are adenocarcinomas consisting of epithelial cell-derived tumor cells [64], suggesting that the CD73 expressed in MSCs has a distinct function. Clarifying the function of CD73 in MSCs is essential for the clinical application of CD73^+^ cells. Finally, the interactions of transplanted CD73^+^ cells with intestinal fibroblasts and other cells are still being determined. Epithelial and immune cells, such as macrophages and neutrophils, have an essential role in an injured site [65]. Single-cell transcriptome analysis in colon tissues after the trans-anal transplantation of CD73^+^ cell spheroids could reveal the interactions between engrafted cells and the tissue-resident cell population.

## 5. Conclusions

We demonstrated an appropriate transplantation method based on the characteristics of a uniform CD73^+^ cell population, and revealed the interaction between the transplanted cells and tissue-resident fibroblasts as one of the mechanisms underlying colitis repression (Figure 7A,B). Our work opens new avenues for treating UC and other inflammation-based diseases. Our findings suggest a potential role for CD73^+^ cell spheroids as a source of transplanted cells or secreted factors in the clinical application of MSCs.

## Data Availability

mRNA-seq data supporting the findings of the present study have been deposited in the Gene Expression Omnibus SuperSeries record GSE211637 (https://www.ncbi.nlm.nih.gov/geo/query/acc.cgi?acc=GSE211637 (accessed on 17 August 2022)).

## Supplementary Materials

The following supporting information can be downloaded at: https://www.mdpi.com/article/10.3390/pharmaceutics15030845/s1, Figure S1: Representative FACS plots of isolation of adipose-derived CD73^+^ cells from mouse subcutaneous fat; Figure S2: Representative FACS plots of isolation of CD73^+^ cells from human adipose tissue; Figure S3: Transplanted cell engraftment by intravenous injection; Figure S4: Improvement of trans-anal transplantation method; Figure S5: 3D culture improves engraftment of CD73^+^ cells into colonic tissue after DSS treatment; Figure S6: Protein expression of MSC markers under 3D culture determined using FACS analysis; Table S1: Mouse qPCR primer sequences used in the present study.
