# Supplementary material for: CD73-Positive Cell Spheroid Transplantation Attenuates Colonic Atrophy
Source: Pharmaceutics. 2023 Mar 4;15(3):845. doi: 10.3390/pharmaceutics15030845 (PMC10051511; doi:10.3390/pharmaceutics15030845)
Supplement: Supplementary file 1 [file pharmaceutics-15-00845-s001.zip › pharmaceutics-2174943-supplementary.pdf]

Supplementary Materials: CD73-Positive Cell Spheroid Transplantation Attenuates Colonic Atrophy

Daisuke Hisamatsu, Natsumi Itakura, Yo Mabuchi, Rion Ozaki, Eriko Grace Suto, Yuna Naraoka, Akari Ikeda, Lisa Ito, and Chihiro Akazawa

Supplementary Figures

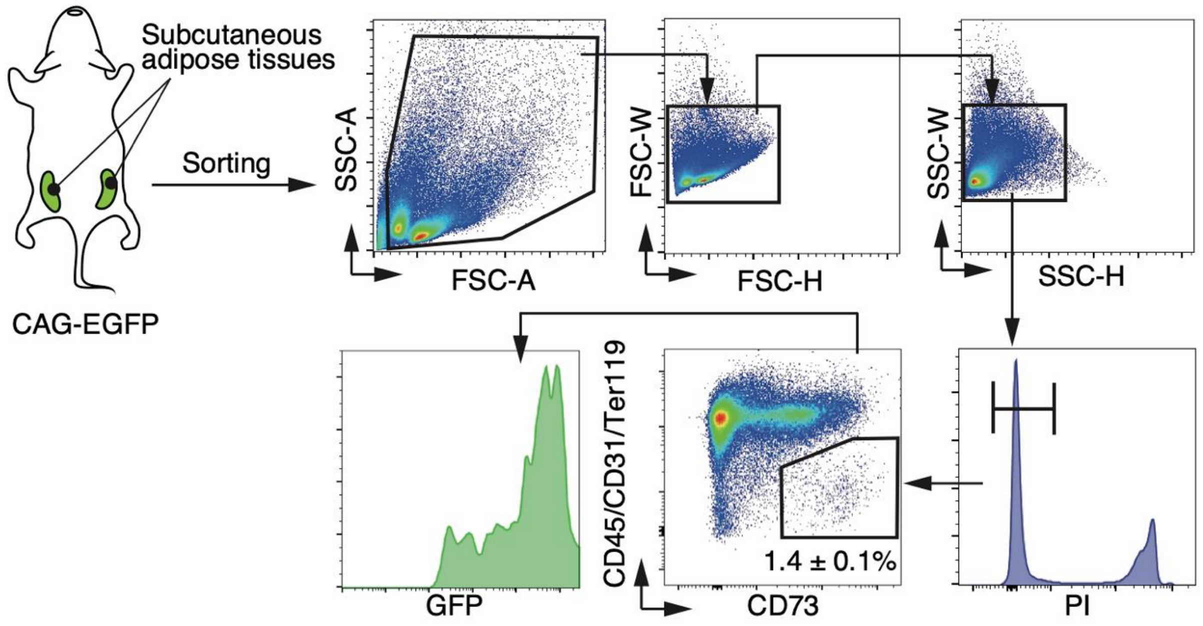

Figure S1. Representative FACS plots, representing the isolation of adipose-derived CD73⁺ cells from mouse subcutaneous fat (*n* = 3 mice).

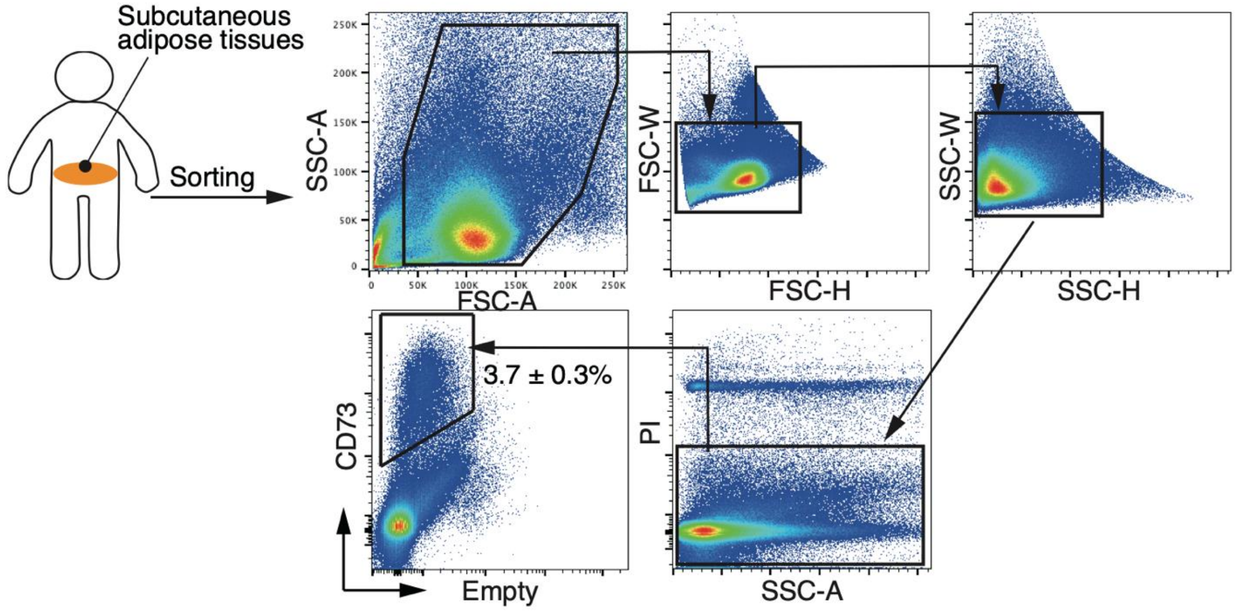

Figure S2. Representative FACS plots, representing the isolation of CD73+ cells from human adipose tissue (n = 3 specimens).

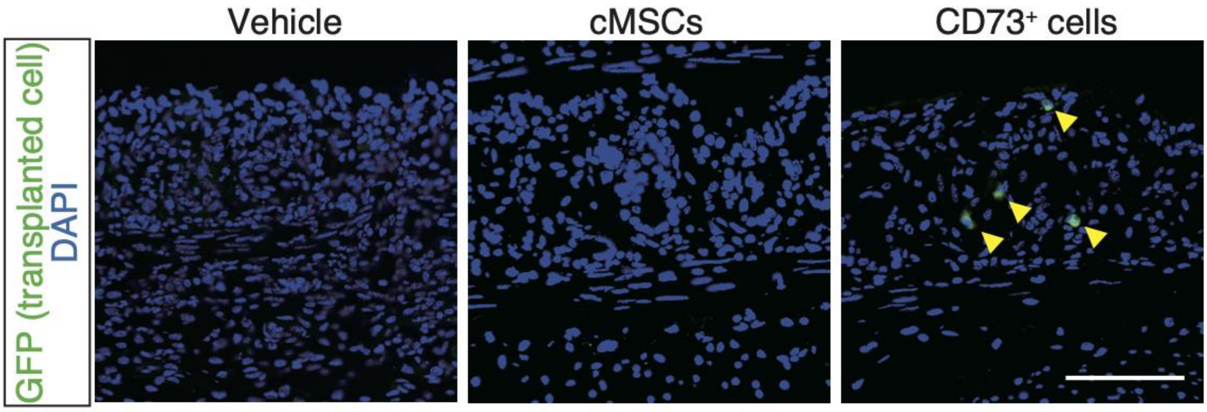

Figure S3. Transplanted cell engraftment by intravenous injection. Representative immunohistochemistry images of GFP$^+$-transplanted cells after intravenous injection. Arrowheads indicate transplanted cells. No cell engraftment was observed in the PBS-treated and cMSC-transplanted groups. Vehicle: PBS-treated group; cMSCs: cMSC-transplanted group; CD73$^+$ cells: CD73$^+$ cell-transplanted group. Scale bar: 100 μm.

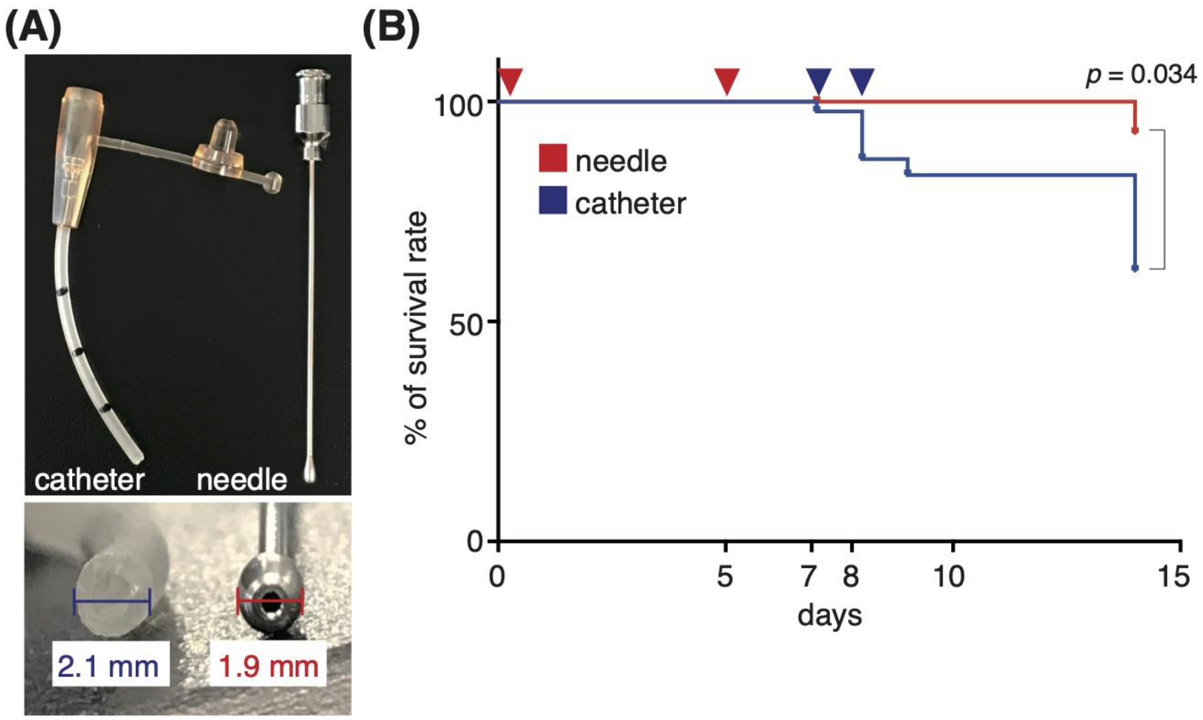

Figure S4. Improvement of trans-anal transplantation method. (A) Comparison of a thin, flexible catheter and a stainless steel needle used for cell injection. (B) Survival rate for each method. n = 5–6 mice. Arrowheads indicate transplantation day. Statistical significances were determined using Welch's t-test.

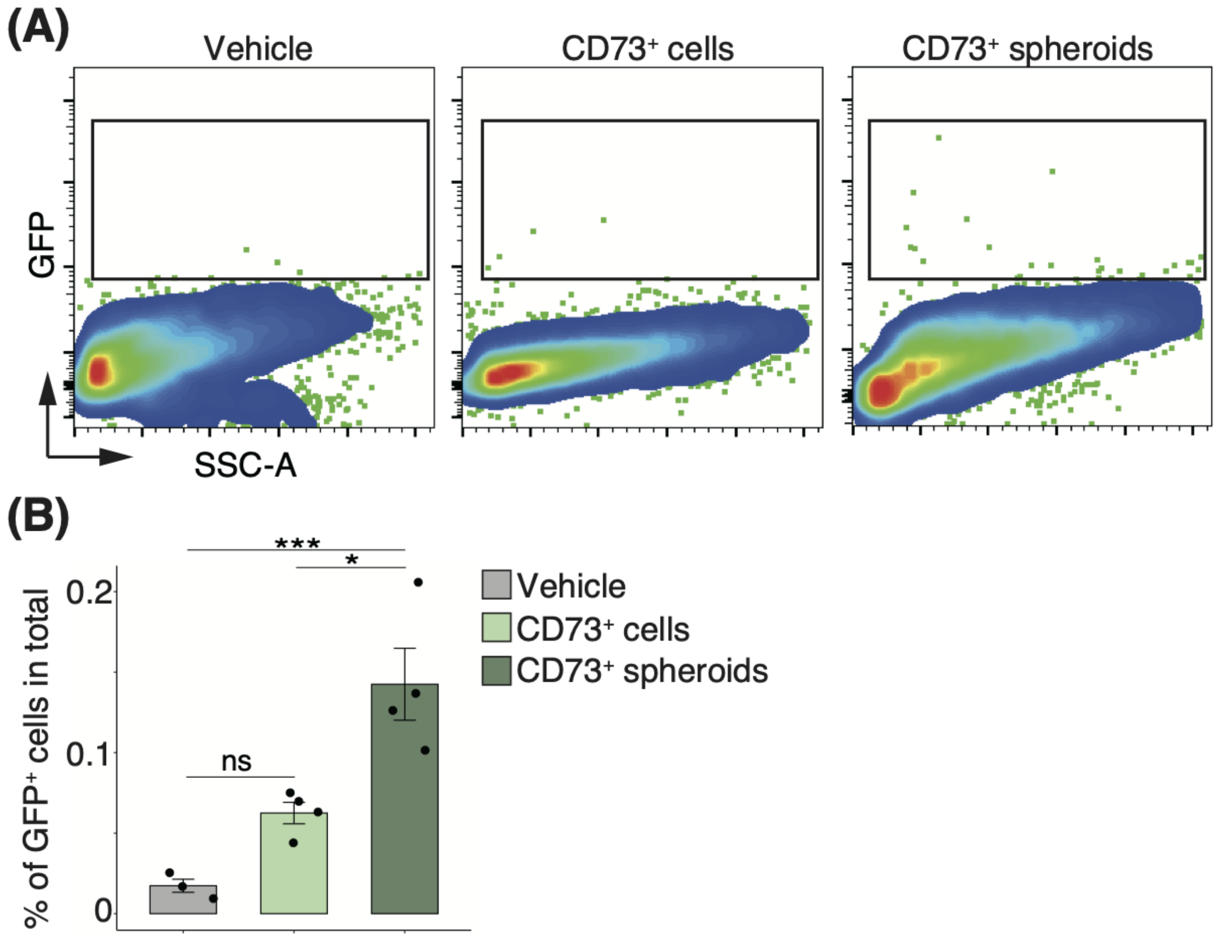

Figure S5. 3D culture improves the engraftment of CD73⁺ cells into colonic tissue after DSS treatment. (A) Representative FACS plots of GFP⁺-transplanted cells after trans-anal transplantation, using a stainless steel needle. (B) Graph showing the percentage of GFP⁺-transplanted cells. Vehicle: PBS-treated group; CD73⁺ cells: CD73⁺ 2D cell-transplanted group; CD73⁺ spheroids: CD73⁺ cell spheroid-transplanted group. n = 3–4 mice. Statistical significances were determined using the Tukey's test. Data are shown as mean ± SEM. $*p < 0.05$; $***p < 0.005$; ns, not significant.

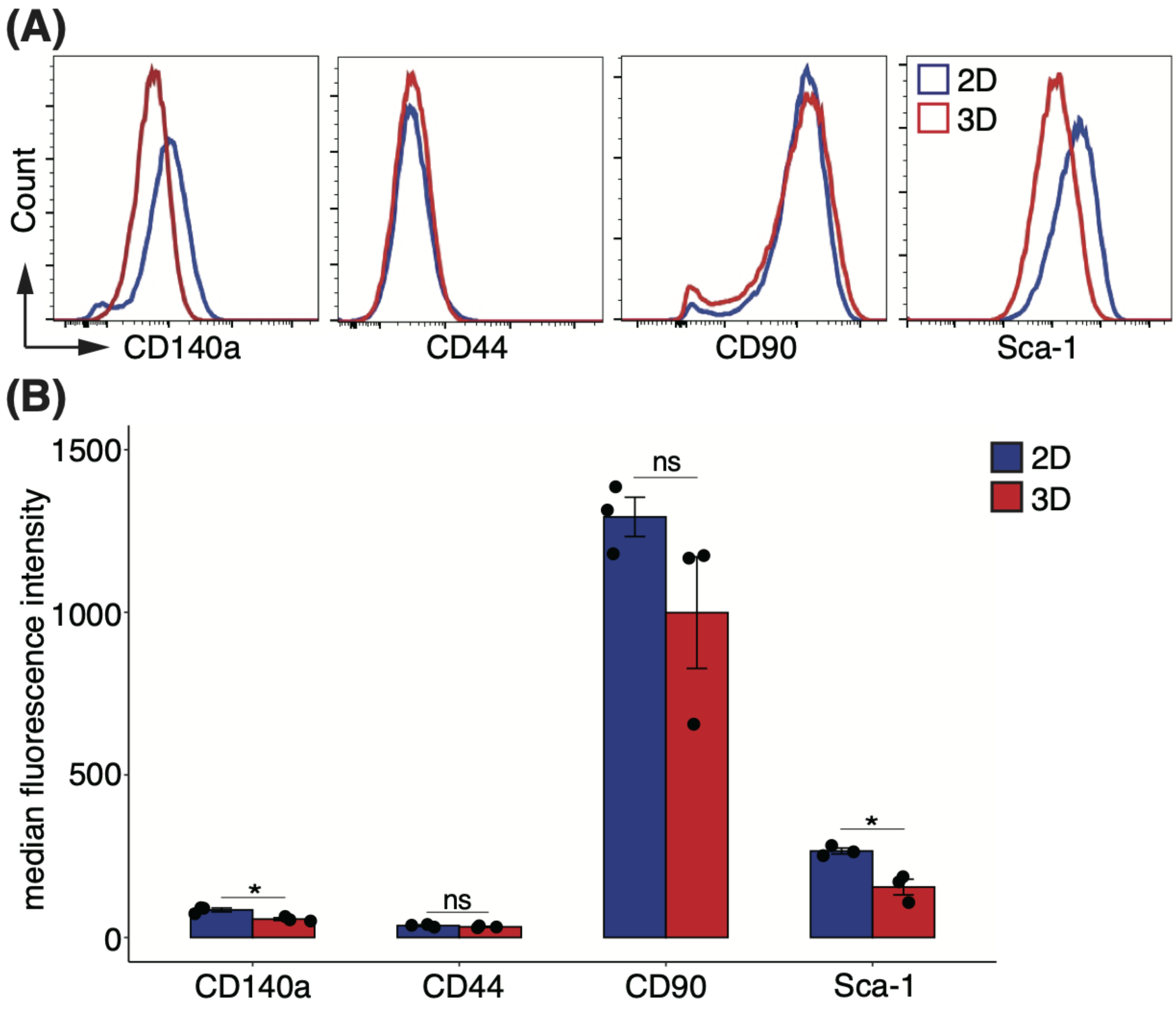

Figure S6. Protein expression of MSC markers under 3D culture, determined using FACS analysis. (A) Representative histograms of each MSC marker in CD73⁺ 2D cells and 3D spheroids. (B) Graph showing the mean fluorescent intensity of MSC markers. $n = 4$. Statistical significances were determined using Welch's t-test. Data shown as mean ± SEM. $*p < 0.05$. ns, not significant.

Table S1. Mouse qPCR primers sequences used in the present study.

Gene	Forward	Reverse
Ago2	TCCCAGGATATGCCTTCAAA	TAAAGTGCTGGACCATGTGC
Axin2	AGAAGCGACCCAGTCAATCC	TTACTCCCCATGCGGTAAGG
Bmp-2	TAGATCTGTACCGCAGGCACTC	CTGCAGATGTGAGAAACTCGTC
Col1a1	ATGTTCAGCTTTGTGGACCT	CAGCTGACTTCAGGGATGT
Col4a1	ATTGTGGTGGCTCTGGCTGT	GCCAGGAAGTCCAGGTTCTC
Ctgf	CCACCCGAGTTACCAATGAC	GCTTGGCGATTTTAGGTGTC
Cxcl1	CAGCCACCCGCTCGCTTCTC	AGGGAGCTTCAGGGTCAAGG
CyclinD1	TGGTGAACAAGCTCAAGTGG	TGGAAAGAAAGTGCGTTGTG
Egf	CCCGAACTTCTCAAACAGAGG	CCATCAGGAAGCAGAACAAATC
Fbxo22	GACAGTTCTCTACATGGCAGATT	ACAATTCCCGGGGTCACA
Fgf-1	AAAAGCCCAAACTGCTCTACTG	GTATAAAAGCCCTTCGGTGTCC
Fgf-2	AAGCGGCTCTACTGCAAGAAC	CATAGCAAGGTACCGGTTGG
Fn1	GCTTTGACAAATACACTGGGAAC	CATTTCCCAGACACAGACACTC
Gadd45a	GCAGAGCAGAAGACCGAAAG	GCAGGCACAGTACCACGTTA
Gapdh	AAAGGGTCATCATCTCCGCC	CTCGTGGTTCACACCCATCA
Hgf	AGTGTGCCAACAGGTGTATCAG	GTCCCTTTATAGCTGCCTCCTT
Icam-1	GAGAGTGGACCCAACTGGAA	GGGTGAGGTCCTTGCCTACT
Igf-1	CAGCTGGACCAGAGACCCTTT	ATCACAGCTCCGGAAGCAAC
Il-6	GGATACCACTCCCAACAGACC	TCCAGTTTGGTAGCATCCATC
Il-8	TCAAGAGCTACGATGTCTGTGT	GGCCAACAGTAGCCTTCACC
Itgb1	CCTTCAATTGCTCACCTTGTTC	CGATGATTAGCTGGATCACATTAC
Itgb3	CCACACGAGGCGTGAACTC	CTTCAGGTTAC ATCGGGGTGA
Itgb5	GAAGTGCCACCTCGTGTGAA	GGACCGTGGATTGCCAAAGT
Itgb7	ACCTGAGCTACTCAATGAAGGA	CACCGTTTTGTCCACGAAGG
Itgb8	CTGAAGAAATA CCCCGTGGA	ATGGGGAGGCATACAGTCT
Lef1	GTCAGACAAGCCCCGTCCT	GCTGTTCATATTGGGCATCATT
Mgp	GCTCCCTCTGGCCATCCT	GCTTTAGCTCGCCACCTCT
Mmp2	ACCAGAACACCATCGAGACC	TACTTTTAAGGCCCGAGCAA
N-myc	TCCTGGGAACTGGGTTGGAG	GACCGCCGAAGTAGAAGTCA
Nox4	CTTTTCATTGGGCGTCCTC	GGGTCCACAGCAGAAAACTC
Paxillin	ACTACTGCAACGGACCCATC	TAGTGGACCTCACAGTACGG
Pdgfb	CTCCATCCGCTCCTTTGATG	GATCGATGAGGTTCCGAGAGA
Postn	CTCAGCACTACTCCGATGTCTC	TTAACCATGTGGCTGTGTAAGG
Rankl	AAACGCAGATTTGCAGGACT	ACATCCAACCATGAGCCTTC
Runx2	GCGTCAAACAGCCTCTTCAG	GTTGTTGCTGTTGCTGTTGT
S100a13	TGGTCTCTACTTTCTTCACCTTTG	TCACATCCAAGGTCTTCATCTTT
Slc3a2	CAGCTCCTACCTGTCAAATTCC	TGGTAGAGTCGGAGAAGATGGT
Smad4	AGGACATTCGATTCAAACCATC	TTTCAAAGTAAGCAATGGAGCA
Sox9	AAGAACAAGCCACACGTCAA	CGTTCTTCACCGACTTCCTC
Tcf7	ATCCTTGATGCTGGGATCTG	CTTCTCTGCCTTGGGTTCTG
Tgf-β1	GTCAGACATTCGGGAAGCAGT	GGTCAGCAGCCGGTTACCAAG
Tsg-6	CTGGCAGATACAAGCTCACCTAC	GGGTATCCGACTCTACCCTTG
Vegfa	CCATGAACTTTCTGCTCTCTTG	GTAGCTTCGCTGGTAGACATCC
Vinculin	GCCAAGCAGTGCACAGATAA	TTCCTTTCTGGTGTGTGAAGC
Wnt11	CTGCACCTCTGGCGACCT	CAGAAGTCAGGGGAGCTCTGT
Wnt3	AGCGTAGCAGAAGGTGTGAAG	GTGGCCCCTTATGATGTGAGT
Wnt5a	GTCCTTTGAGATGGGTGGTATC	ACCTCTGGGTTAGGGAGTGTCT
Wnt5b	CCCCAGGCCAGAGAAAGC	CCTCCCCGATGTAGGACAT
